# Research Progress of Wide Tunable Bragg Grating External Cavity Semiconductor Lasers

**DOI:** 10.3390/ma15228256

**Published:** 2022-11-21

**Authors:** Xuan Li, Junce Shi, Long Wei, Keke Ding, Yuhang Ma, Kangxun Sun, Zaijin Li, Yi Qu, Lin Li, Zhongliang Qiao, Guojun Liu, Lina Zeng, Dongxin Xu

**Affiliations:** Key Laboratory of Laser Technology and Optoelectronic Functional Materials of Hainan Province, Academician Team Innovation Center of Hainan Province, College of Physics and Electronic Engineering, Hainan Normal University, Haikou 571158, China

**Keywords:** tunable, narrow linewidth, Bragg grating, BG-ECSL

## Abstract

In this paper, we review the progress of wide tunable Bragg grating external cavity semiconductor lasers (BG-ECSLs). We concentrate on BG-ECSLs based on the wide tunable range for multicomponent detection. Wide tunable BG-ECSLs have many important applications, such as wavelength-division multiplexing (WDM) systems, coherent optical communications, gas detection and atom cooling. Wide tunability, narrow linewidth and a high side-mode suppression ratio BG-ECSLs have attracted much attention for their merits. In this paper, three main structures for achieving widely tunable, narrow linewidth, high side-mode suppression ratio BG-ECSLs are reviewed and compared in detail, such as the volume Bragg grating (VBG) structure, fiber Bragg grating (FBG) structure and waveguide Bragg grating (WBG) structure of ECSLs. The advantages and disadvantages of different structures of BG-ECSLs are analyzed. The results show that WBG-ECSLs are a potential way to realize the integration, small size, wide tuning range, stable spectral output and high side-mode suppression ratio laser output. Therefore, the use of WBG as optical feedback elements is still the mainstream direction of BG-ECSLs, and BG-ECSLs offer a further new option for multicomponent detection and multi-atoms cooling.

## 1. Introduction

ECSLs usually refer to the gain chip based on the introduction of external optical components (such as waveguides, gratings, prisms, etc.) to provide optical feedback. By designing the type, position and structure of external optical components, the optical properties of SLs (such as center wavelength, linewidth, tuning range, side-mode suppression ratio (SMSR), etc.) can be changed [1,2,3]. BG-ECSL [4] is a device with a specific optical element (Bragg grating) in the external cavity. BG-ECSLs have excellent performances, such as narrow linewidth, tunability and high SMSR. They are widely used in WDM systems [5,6,7], coherent optical communication [8], gas detection [9], Lidar [10], atomic physics [11] and other fields. In this paper, three main structures for achieving widely tunable, narrow linewidth, high side-mode suppression ratio BG-ECSLs are reviewed and compared in detail, such as volume Bragg grating (VBG) structure, fiber Bragg grating (FBG) structure and waveguide Bragg grating (WBG) structure of ECSLs. The advantages and disadvantages of different structures of BG-ECSLs are analyzed. Through comparative analysis properties of BG-ECSLs, WBG-ECSLs have significant advantages over other BG-ECSLs in terms of structure design, functional types and application fields. BG-ECSLs will offer a further new option for multicomponent detection and multi-atoms cooling in the future. In recent years, with the continuous development of WDM system, gas detection and other fields, BG-ECSL will be applied in more fields and gradually become a research hotspot in the field of SLs.

## 2. Principle of BG-ECSLs

Grating, also known as diffraction grating, is an optical element composed of a large number (tens of thousands) of equally wide and equally spaced slits, which can make the amplitude or phase of the incident light, or both, produce periodic spatial modulation.

**Figure 1:**
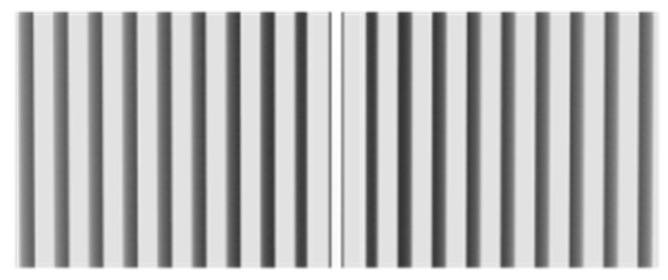
Structure of Bragg grating.

Bragg gratings (period less than 1 μm, also known as reflection gratings) are transparent devices with a periodically varying refractive index. Their structure is shown in Figure 1. The reflectance is large in the wavelength region (bandwidth) near a particular wavelength, which satisfies the Bragg condition:

mλ_B_ = 2n_eff_Λcosθ (1)

In Formula (1), m is the diffraction order, λ_B_ is the Bragg wavelength, n_eff_ is the effective refractive index of the medium, Λ is the grating period and θ is the propagation angle in the medium relative to normal incidence.

**Figure 2:**
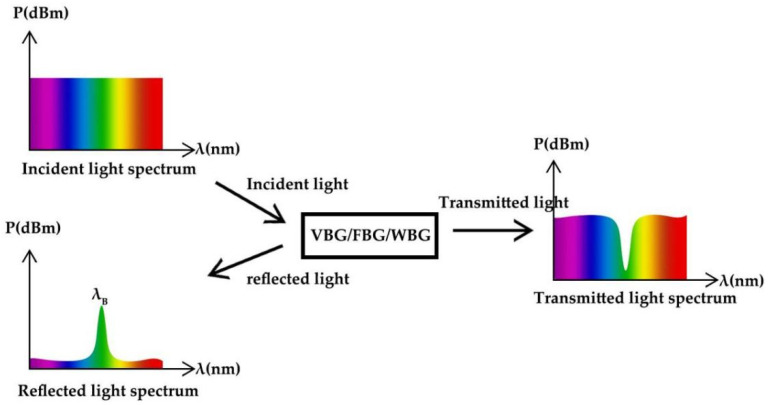
Bragg grating reflection principle.

If the above conditions are met, the difference between the wave number of the grating and the wave number of the incident and reflected waves is matched. Other wavelengths of light are hardly affected by the Bragg grating but still produce some sidelobes in the reflection spectrum. Similarly, there is almost no reflection of the beam at other angles of incidence. When the grating is long enough, even a very weak refractive index modulation can achieve almost total reflection of the beam near Bragg wavelength. The principle is shown in Figure 2.

The linewidth (∆ν) of BG-ECSLs (linewidth is usually used to quantitatively characterize the spectral purity of its temporal coherence) can be expressed as follows:

(2) $$\Delta v=(1+\alpha^{2})\frac{{\upsilon_{g}}^{2}h\nu gn_{sp}\alpha_{m}}{8\pi P_{0}}$$

In Equation (2), *α* is the specific linewidth increase factor of the semiconductor laser, *υ_g_* is the group velocity, h is the Planck constant, *ν* is the frequency, g is the gain factor, *n_sp_* is the spontaneous emission factor (reflecting incomplete particle number inversion), *α_m_* is the output loss of the cavity and *P*_0_ is the output power.

Where α can be expressed as:

(3) $$\alpha =\frac{d\vec{n}/\mathrm{dN}}{\mathrm{dg}/\mathrm{dN}}$$

vector n and g represent the real and imaginary parts of the complex refractive index of the active medium, respectively, and dg/dN is the differential gain. Due to the different materials, it is generally between 2 and 5, and from Equation (2), it can be seen that the output power of the laser is inversely proportional to the linewidth.

The SMSR of BG-ECSLs (SMSR is usually a replay index used to characterize its single-longitudinal modeling) can be expressed as:

(4) $$SMSR=10\mathrm{lg}\frac{P_{1}}{P_{2}}$$

where *P*_1_ is the optical power of longitudinal mode, and *P*_2_ is the maximum optical power of edge mode. The larger the SMSR of the laser, the better its single-mode characteristics, and the more stable the single-mode output of the laser. When the edge mode rejection ratio is greater than 20 dB—that is, the optical power of the main longitudinal mode is more than 100 times of the maximum optical power of the edge mode—the laser in this working state can be considered as a single longitudinal mode laser.

## 3. Research Progress of BG-ECSLs

The structures of BG-ECSLs can be mainly divided into the VBG structure, FBG structure and WBG structure. This paper mainly discusses the research progress of VBG structure, FBG structure and WBG structure of BG-ECSLs. BG-ECSLs have a long history. Since the 1970s, in order to make Bragg grating in fiber and other materials, people have carried out a lot of research on the effect of UV radiation damage in silica, germanium-doped silicon glass and other materials. Nowadays, there are many kinds of Bragg grating, and the application fields are also very wide. Due to the unique characteristics and potential application prospects of Bragg grating, since the first batch of BG-ECSLs came out, a large number of companies have studied and continuously optimized the performance of BG-ECSLs. At present, Coherent, OptiGrate, DILAS and so on are well-known in the world. Among them, the United States, Germany, France, Sweden, Canada, Japan and other countries have certain technical advantages in the development of BG-ECSLs. In China, there are also a large number of companies and research institutions developing relevant cutting-edge technologies, such as Wuhan National Laboratory of Optoelectronics, Shanghai Institute of Optics and Precision Instruments, Chinese Academy of Sciences, Beijing Institute of Semiconductors, Chinese Academy of Sciences, Changchun Institute of Optics, Fine Mechanics and Physics, Chinese Academy of Sciences, etc. BG-ECSLs can be widely used in optical communication, biotechnology, environmental detection, material processing and other fields and has potential application prospects in high-precision detection, quantum communication system and other fields.

### 3.1. VBG-ECSLs

**Figure 3:**
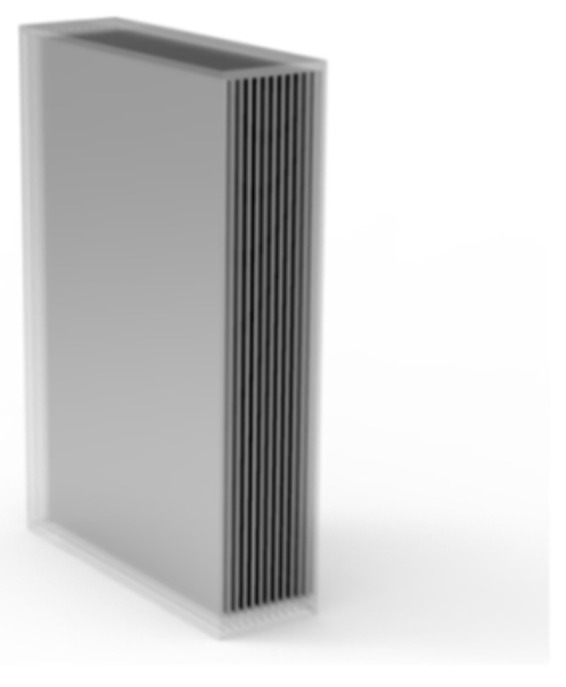
Structure of VBG.

VBG is a holographic optical element recorded in photothermal refraction glass, which is an effective spectral and angular filter capable of withstanding high-power laser radiation. Its structure is shown in Figure 3. VBG can be divided into reflector volume Bragg grating (RVBG), transmission volume Bragg grating (TVBG) and chirped volume Bragg grating (CVBG). A VBG operating in reflective geometry is called an RVBG when the diffracted beam passes through the grating surface directly toward the incident beam. When the diffracted beam passes through the rear surface of the grating, the VBG operating in the form of transmission geometry is called TVBG. When the period of VBG changes gradually along the direction of beam propagation, it is called CVBG. The RVBG is similar to a narrowband spectrum filter, while the TVBG is similar to a narrowband angle filter.

VBG can not only be used to lock the wavelength but also enhances the oscillation of the main lobe and suppresses the side lobe, so as to reduce the beam divergence and improve the beam quality. The biggest advantage of VBG compared to FBG and WBG is that it can change the angle of incidence of light and, thus, its optical properties.

**Figure 4:**
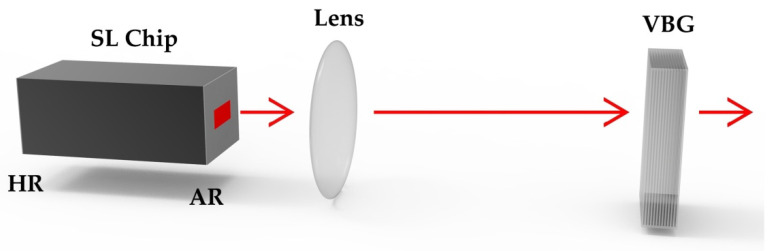
Structure of VBG-ECSL.

The structure of VBG-ECSL is shown in Figure 4, which mainly includes SL chip, VBG and other optical components. By adjusting the incidence angle, VBG diffraction rate, injection current and other operations, the reflection wavelength can be changed to reduce the loss, so as to optimize the performances of VBG-ECSL. Compared with other ECSL, VBG-ECSL can achieve higher resonant feedback, so as to narrow the spectrum of the SL emission without significantly reducing the power and efficiency.

In 2010, Daghestani et al. [12] reported stable dual-wavelength operation by combining a single VBG with a quantum dot-based gain medium in a compact external cavity structure. The dual-wavelength outputs were concentrated at 1179.9 nm and 1182.8 nm, with a spectral bandwidth of 0.2 nm for each mode. At the operating current of 1 A, the maximum output power reached 186 mW, and the SMSR of both modes was greater than 34 dB. The slope efficiency was 0.26 W/A, and the linewidth was 0.35 nm.

In 2011, Li Shen et al. [13,14] reported a novel electro-optic tuning VBG-ECSL with VBG. A high electro-optical coefficient lead lanthanum zirconate titanate (PLZT) transparent ceramic was inserted into the external cavity as a refractive index adjustable element to change the cavity length. A single-mode tuning range of 2.5 GHz was achieved by adjusting the voltage applied to the PLZT. The center wavelength was about 810.0 nm, the linewidth was 19 MHz and the SMSR was 37 dB. The combination of VBG and PLZT transparent electro-optic ceramics allowed the fine and rapid tuning of laser frequencies over a range of longitudinal mode spacing. In addition, the electrode form of PLZT ceramic was designed to change the angle of the incident beam, so as to achieve 32.9 GHz (810.03~810.102 nm) tuning.

In 2012, B. Sempf et al. [15] reported a tunable miniaturized 633 nm VBG-ECSL. The structure contained no moving parts and only changed the injected current to tune the ECSL wavelength. The slope efficiency was 0.3 W/A, the linewidth was less than 10 MHz, the SMSR was more than 25 dB and the maximum output power reached 10 mW. Tunable continuous range was 34 pm.

In the same year, Yufeng Li et al. [16] reported the wavelength locking of high-power VBG-ECSL. It was found that using VBG with high reflectance was easier to achieve wavelength locking, but the output power and service life of the device would be reduced.

In 2013, E Luvsandamdin et al. [17] reported a microintegrated VBG-ECSL module to implement a microintegrated, narrow linewidth VBG-ECSL for an accurate spectral analysis of ultra-cold rubidium atoms on line D2 (780.24 nm). The microintegrated VBG-ECSL consisted of a laser chip, a volume holographic Bragg grating (VHBG) and a pair of aspheric lenses. Separate temperature control of the VHBG was achieved by a miniature thermoelectric cooler for coarse wavelength tuning. The laser achieved a power output of 123 mW at a single-mode operation of 780.24 nm at the injection current of 250 mA. The slope efficiency is 0.88 W/A, the linewidth was about 2 kHz and the SMSR was more than 57 dB. The coarse tuning range was 44 GHz and continuous tuning range was 31 GHz.

In 2014, Luvsandamdin et al. [18] reported a microintegrated VBG-ECSL module. The module consisted of a laser chip, a collimating lens, a low-light level isolator, a miniature temperature sensor and a miniature thermoelectric cooler with VHBG. It emitted at a wavelength of 766.7 nm and could be continuously tuned at 27.5 GHz. At an injection current of 240 mA, and a output power of 33.4 mW was provided. The linewidth was 3 kHz, the SMSR was greater than 45 dB and the slope efficiency was 0.40 W/A.

In 2015, Rauch et al. [19] reported an easy-to-use, compact package VBG-ECSL. The VBG-ECSL had a power output of up to 380 mW at an operating wavelength of 780 nm and mainly included a thermoelectric cooler, laser chip, collimating lens and bulk holographic grating (VHG). The linewidth was 36 kHz, the SMSR was greater than 50 dB and the tunable range was 10.5 pm (5.2 GHz) continuously.

In the same year, Zheng et al. [20] reported an VBG-ECSL. VBG was used as the output coupler to further stabilize the wavelength and narrow the linewidth. The laser had a center wavelength of 1064 nm and a continuous tuning range of 110 pm. At the injection current of 3 A, the linewidth was reduced from 80 pm to 33 pm, and the output power reached 1.3 W.

In 2016, Ruhnke et al. [21] proposed a compact VBG-ECSL module with a narrow linewidth emission of 1.4 W at 445 nm. The module used a GaN-based SL as the gain medium and integrated a VBG for wavelength stabilization. The linewidth was about 1 nm, and the SMSR was greater than 50 dB. When the injection current was 1.2 A, the output power was 1.4 W, and the slope efficiency was 1.3 W/A. In the same year, Ivanov et al. [22] used the VHBG to select the longitudinal mode of VBG-ECSL. The emission spectrum width of the laser was reduced from 6 nm to 0.5 nm. Kohfeldt et al. [23] reported a VBG-ECSL based on VHBG. The center wavelength of the laser was 767 nm, and the linewidth was 55 kHz. The continuously tunable range was 50 GHz. At an injection current of 200 mA, the output power exceeded 30 mW.

In 2017, Heike Christopher et al. [24] reported a high-power VBG-ECSL module with a narrow linewidth. The output wavelength of the module was 1064.49 nm, which was composed of a SL chip, VHBG, optical isolator and other devices. At an injection current of 100 mA, the output power was 4 mW. The SMSR was larger than 45 dB, and the linewidth was 30 kHz.

In 2018, Zheng et al. [25] reported continuous wave dual wavelength VBG-ECSL, which was used a distributed feedback laser with a single external cavity by using VBG. At an injection current of 2.5 A and operating temperature of 29.8 °C, the output power reached 415 mW, and the tunable spectral difference between the two wavelengths reached 1.92 nm (0.88 THz). The linewidth was 0.27 nm, and the SMSR was 26 dB. In the same year, Ruhnke et al. [26] reported a compact 222.5 nm VBG-ECSL. A microintegrated GaN-based ECSL module with wavelength stable VBG was used as the pump source to generate narrow linewidth deep ultraviolet radiation in β-Bab_2_O_4_ (BBO) crystal. The output power was 160 μW at 1.4 W pump power.

In 2019, Chung et al. [27] reported two second-order PQ:PMMA (phenquinone:polymethyl methacrylate) reflection VBGs with Bragg wavelengths of 488.8 nm and 525.6 nm, respectively, using 532 nm as the recording wavelength. The 525.6 nm VBG was successfully used as the ECSL cavity mirror for the 522 nm SL, and the output spectrum was narrowed by more than 10,000 times. In the same year, Fei Wu et al. [28] reported a 780 nm narrow linewidth VBG-ECSL. The linewidth of the laser was 100 kHz, and the output power is 50 mW.

In 2020, Sin Hyuk Yim et al. [29] reported a 780.25 nm VBG-ECSL. The design allowed the external cavity configuration to be adjusted by adjusting the VHG, with a continuous tuning of 8 GHz and a linewidth of 19 kHz. The output power was 30 mW at an injection current of 150 mA.

In the same year, Mingjun Chi et al. [30] reported 808 nm microintegrated high-power VBG-ECSL. A peak output power of 4.3 W was obtained at an injection current of 6 A.M. Rådmark et al. [31] reported a novel 785 nm VBG-ECSL. A high-reflection VBG element was used to obtain a narrow general linewidth and a very high SMSR.

In 2021, Bin Liu et al. [32] reported a high SMSR and high-stability VBG-ECSL array, which was used VBG as the optical feedback. The central wavelength was 976 nm, the diffraction efficiency was 15% and the linewidth was 0.5 nm. At an injection current of 50 A, the output power was 33.9 W, and the SMSR was greater than 30 dB.

In 2022, Liu et al. [33] reported a wavelength 405.1 nm VBG-ECSL. The spectral linewidth of the VBG-ECSL was 0.08 nm, and the output power was 292 mW. Yuhuan Lu et al. [34] reported a high-power tunable dual-wavelength composite external cavity structure using VHG and VBG. The adjustable frequency difference of the dual wavelength output was between 0.41 THz and 3.89 THz. When the frequency difference was 1.86 THz, the output power was 2.1 W. The SMSR was greater than 29 dB over the entire tunable dual-wavelength output range.

In the same year, Chi et al. [35] designed a microintegrated VBG-ECSL. The VBG-ECSL had a linewidth of 4 pm, the SMSR was about 16 dB and continuous tuning range was 12 pm. Qi et al. [36] reported a miniature integrated narrow linewidth VBG-ECSL based on a VHBG around 780 nm. VBG-ECSL consisted of a gain chip, micro-optics lens, VHBG, optical isolator and thermistor. The ECSL had a linewidth of 70 kHz, the output power was 30 mW and continuously tunable range was 9 GHz. Wang et al. [37] reported an VBG-ECSL. More than 100 W of continuous power output was generated for metastable argon atomic pumping with a slope efficiency of 12.23 W/A. The laser had a linewidth of 0.15 nm, a tunable range of more than 200 pm and a SMSR of up to 40 dB.

In addition to universities and research institutions, VBG-ECSLs has been commercialized by a number of Photonics companies, including Thorlabs of the United States, Coherent’s Ondax and DILAS of the United States, RPMC of the United States and Photontec of Germany.

Coherent, Inc., founded in May 1966, is one of the world’s photonics manufacturers and innovators. The company designs and produces many of its own components and subcomponents, including fiber optics, crystals and wafer growth. Coherent has a wide range of products, including gas, fiber, solid-state and semiconductor lasers, and is a major photonics company in the United States. Among other things, it has acquired DILAS, Ondax and others.

DILAS is a global leader in high-power semiconductor laser technology and the world’s largest high-power semiconductor laser company. Its products are widely used in industrial processing, laser medicine, information display, national defense and scientific research and other fields, and the product wavelength range is 635~1940 nm. The company’s VBG laser designs include central wavelengths of 790 nm, 808 nm, 976 nm and 981 nm, with primary power outputs ranging from 20 W to 120 W and up to 240 W. Take the 808 nm VBG semiconductor laser with the output power of 40 W as an example, the laser can be continuously tuned to 1.2 nm, the output power can reach 40 W, the linewidth is less than 0.5 nm and the wavelength drift coefficient is 0.01 nm/°C. It can be seen that the output of this kind of product is stable, and the power is extremely high.

Ondax is a professional supplier of wavelength stabilized semiconductor lasers for volumetric holographic gratings and related applications, including: 405 nm, 638 nm, 780.25 nm and 808 nm wavelength stabilized semiconductor lasers; collimating wavelength stabilized semiconductor lasers; wavelength stabilized Semiconductor Butterfly lasers and fiber coupled laser modules. Ondax offers a variety of products, such as the SureLock family of products, all of which are stabilized using VBG to ensure accurate, ultra-stable output wavelengths. The main center wavelengths of the series are 785 nm, 808 nm, 830 nm, 976 nm and 1064 nm; the linewidth is 0.1 nm; the SMSR is 40 dB and the output power is over 800 mW. The advantages of this product are high output power and small linewidth. Moreover, the wavelength flowing with the temperature is very small, only 0.01 nm/°C, which can be applied to the fields of measurement and sensing.

RPMC (USA) is a comprehensive semiconductor laser and semiconductor pumped solid-state laser company. It not only designs and produces semiconductor lasers but also distributes more than ten brands of foreign lasers in North America. The linewidth of the 1064 nm VBG laser designed by the company is less than 100 kHz, and the wavelength varies with the temperature only at 0.007 nm/°C, which is suitable for gas sensing, metering and other fields.

Photontec (Germany) was founded in 2010 and is located in Berlin, Germany. It is engaged in the development and manufacturing of Diode-pumped solid-state lasers and fiber-coupled diode laser modules. The company’s designed 976 nm VBG laser has a power output of up to 100 W and a linewidth of less than 1 nm.

Thorlabs (USA) is a rapidly growing photonics manufacturer and distributor of optical–mechanical, optical and optoelectronic devices and equipment. The company’s 785 nm VBG laser has a stable wavelength output suitable for Raman spectroscopy, microscopy and other fields.

The research performances of VBG-ECSLs in recent years are shown in Table 1. In the early stage, VBG is mainly used to lock the output wavelength of high-power lasers, and in the later stage, VBG is mainly used to narrow the general linewidth of low-power lasers and improve the SMSR. Moreover, VBG can achieve a stable dual wavelength output. When using VBG, the tuning mechanism can be simplified to some extent. Compared with other Bragg gratings, one of the advantages of VBG is that the incident angle is not limited, and the output wavelength can be changed by adjusting the incident angle. Moreover, it can be changed by changing the size of the injection current. The thermal stability of VBG is good, and the wavelength deviation with the temperature is small. The temperature coefficient of the peak wavelength of a typical VBG is 0.01 nm/°C, which plays a significant role in the frequency selectivity and stability. As can be seen from Table 1, the linewidths of most VBG-ECSLs developed in the laboratory are in the range of kHz~GHz, the SMSR is greater than 25 dB and the tuning range is in the order of GHz (most of them are tens of pm). The narrowest linewidth can be as low as 2 kHz, while the SMSR is as high as 57 dB, but the continuously tunable range is very small, only 0.063 nm. The maximum tuning range is 1.9 nm, SMSR is 26 dB, but the linewidth is 124 GHz. The linewidths of commercial VBG-ECSLs developed by the company are generally in the order of GHz, from tens of GHz to hundreds of GHz, SMSR is greater than 35 dB and the tuning range is in the order of GHz (most of them are 1~2 nm). The narrowest linewidth is less than 100 kHz but has only 1 nm tuning capability. The maximum tuning range is 2 nm, but the linewidth is 314.9 GHz. Compared with the laboratory research results, the commercial VBG-ECSLs have a larger tuning range and output power but a wider linewidth. VBG-ECSLs generally consist of a SL chip, micro-optical lens, VBG, optical isolator and thermistor. The advantages of the VBG-ECSLs are those where the external cavity is simple and there are no movable parts, thus providing the maximum mechanical stability and reliability, and the center wavelength of the outer cavity structure can be adjusted by tilting the angle of the VBG or changing the cavity length. During the operation of the device, the direction and position of the output beam of VBG-ECSLs remain on the original path, providing good long-term stability. In the case of a constant operating temperature, SMSR is mainly related to the matching between the divergence angle of a laser beam along the fast axis and the receiving angle of VBG. Using higher reflectance and a larger acceptance angle can produce a narrower general linewidth and higher SMSR, but the output power and reliability of the device will be reduced to some extent. Therefore, it is necessary to set the appropriate reflectance and acceptance angle when designing the structure. In general, the wavelength locking of ECSL can be achieved by introducing VBG into the outer cavity. It can not only achieve wavelength locking but also effectively reduce the linewidth of ECSL. The performance of VBG-ECSLs can be improved by changing the incident angle of light, coupling coefficient and VBG tilt angle. At present, the preparation technology of VBG-ECSLs is relatively mature, and it plays an important role in biomedicine, optical data storage, synthetic aperture Lidar, quantum optical sensors and other fields.

### 3.2. FBG-ECSLs

**Figure 5:**
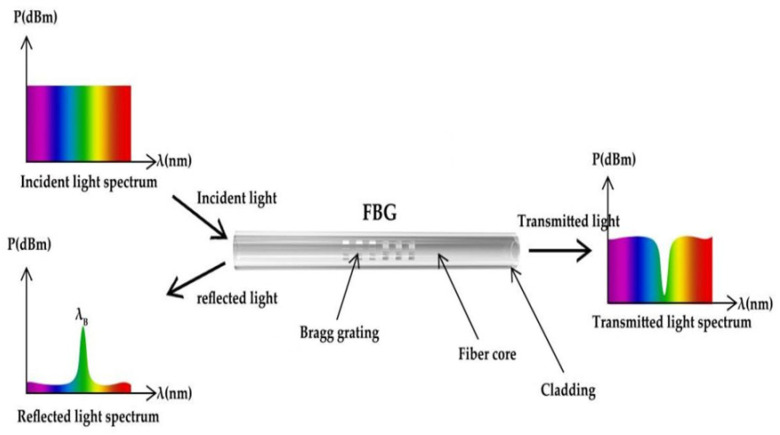
FBG reflection principle.

FBG is a section of grating where the refractive index changes along the axial direction. It has the characteristic of frequency selection, and the light conforming to the Bragg condition is reflected. The reflectance and linewidth of FBG are determined by the period, length, refractive index change and modulation intensity of the grating. The FBG equation is:

λ_B_ = 2n_eff_Λ (5)

where λ_B_ is the Bragg wavelength of the reflection center, n_eff_ is the effective refractive index of the fiber and Λ is the FBG period. When the light of the wide spectral range is an incident on the grating, part of the light continues to propagate forward, and part of the light is reflected. The reflected light has a narrow linewidth, so a narrow linewidth reflection spectrum is generated, as shown in Figure 5.

**Figure 6:**
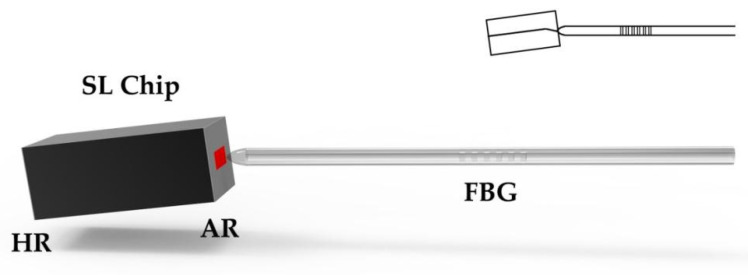
Structure of FBG-ECSL.

FBG-ECSL is a device that couples FBG with a SL chip, and its structure is shown in Figure 6. Since the temperature coefficient of SiO_2_ is much lower than that of the semiconductor, the thermal stability of the device will be improved to a certain extent. By adjusting the temperature of the FBG or external stress, the Bragg wavelength of FBG grating can be changed, so as to realize the tuning function of FBG-ECSL wavelength. Moreover, due to the large group delay characteristic of the bevel edge of the FBG, the linewidth of the ECSL can be effectively narrowed. Moreover, the FBG structure and SL chip can be designed separately, and the coupling angle can be adjusted to reduce the coupling loss, so as to optimize the laser performance. FBG-ECSL is also easy to make a FBG where the linewidth is less than the longitudinal mode spacing of a SL chip, which is beneficial to obtaining a high SMSR.

In 2010, Bretislav et al. [43] reported an FBG-ECSL for interferometry, which was used FBG to improve the wavelength stability and tunability. The central wavelength of the laser was 760 nm, the linewidth was less than 0.4 nm and the laser can be tuned continuously for 1 nm.

In 2011, Huang et al. [44] reported an optimized dual FBG for 980 nm FBG-ECSL, thereby limiting the wavelength shifts due to temperature. A mathematical model of he wavelength shift caused by the temperature was established by using the feedback rate equation of the external cavity, and the parameters of the external cavity were optimized to achieve a stable mode-locked laser output. In the range of 0~70 °C, the SMSR was higher than 45 dB, and the linewidth was less than 1nm. At the current of 400 mA, the output power was greater than 200 mW.

In the same year, Loh et al. [45] reported a 1550 nm high-power, low-noise package InGaAlAs/InP quantum well FBG-ECSL. The laser included a double-pass curved channel plate-coupled optical waveguide amplifier that used lens fibers coupled to a passive cavity of a narrowband (2.5 GHz) FBG. At a current of 4 A, the FBG-ECSL produced 370 mW of fiber-coupled output power with a linewidth of 1kHz and a relative intensity noise of 160 dB/Hz from 200 kHz to 10 GHz.

In 2012, Chen et al. [46] used the structure of an InP SL gain chip and FBG external cavity, where the emission wavelength was consistent with the methane absorption line, which was used for spectral gas sensing. FBG was used as a wavelength selector and feedback element that potentially provided the laser with wavelength tuning capabilities. The laser achieved a narrow linewidth of less than 5 MHz and was the first FBG-ECSL in the 1.65 µm region.

In 2014, Guo et al. [47] analyzed the effect of high SMSR on the ECSL of FBG using coupled cavity theory. A high SMSR and high stable frequency of FBG-ECSL were obtained by experiments. The central wavelength of the laser was 974 nm, the SMSR was 45 dB and the rate of change of the central wavelength reached 3.08 pm/°C in the temperature range of 20~80 °C. The output power was 7.1 mW at a 100 mA current.

In the same year, Teh et al. [48] reported transform-limited laser pulses of about 18 ps from FBG-ECSL. The SMSR of the laser was 50 dB, and the maximum wavelength offset was 0.1 nm. Lynch et al. [49] reported an FBG-ECSL, which used SL gain chips and FBG, where the thermal expansion or mechanical strain provided the wavelength tuning mechanism. The laser had a center wavelength of 1647 nm, a peak reflectivity of 50%, a 3 dB bandwidth of 0.5 nm and a tuning range of 1 nm.

In 2016, Lynch et al. [50] reported an ECSL using a novel integrated fiber platform Bragg grating as the external cavity. The cavity connected the photosensitive fiber to the silica on the silicon wafer and wrote the grating using the direct UV writing method. The laser operated in single mode at the acetylene P13 line (1532.83 nm) with a SMSR greater than 60 dB and linewidth less than 14 kHz. The output power was 9 mW at the injection current of 500 mA.

In the same year, Wei et al. [51] reported a simple and low-cost 1549.7 nm SL with an intrinsic linewidth of sub-kHz. The laser had an output power of 3 mW at an injection current of 131 mA with a continuous tuning range over 0.8 nm and a linewidth of 125 Hz. Wei et al. [52] designed a butterfly package narrow linewidth hybrid integrated ECSL based on polarization preserving FBG. The ECSL emitted at a wavelength of 1550 nm, had a linewidth of less than 3 kHz and had a continuous tuning capability of 21 GHz. The output power was 20 mW at an injection current of 270 mA.

In 2017, Zhang et al. [53] reported a 1550 nm mode-free jump ECSL design that included a SL gain chip and an FBG with enhanced thermal sensitivity. The features of ECSL were a narrow linewidth of 35 kHz and high linear thermal tuning speed of 65 pm/°C (8.125 GHz/°C), which was six times higher than the normal FBG-ECSL. The SMSR was larger than 50 dB, and the continuous mode no-hop tuning range was 0.5 nm, which was five times larger than the cavity mode spacing.

In 2018, Yan et al. [54] reported a theoretical model of FBG using the transfer matrix method. The effects of the FBG length and modulation depth on reflectance, grating spectral width and wavelength were studied. Some 976 nm laser modules with different grating reflectance (4%, 6%, 8% and 10%) were fabricated. By increasing the reflectance of the FBG, the SMSR was optimized to more than 40 dB, and the threshold current was reduced.

In 2019, Yang et al. [55] reported a 1550 nm continuously tunable laser using chirped FBG and a semiconductor optical amplifier (SOA). The linewidth of the laser was less than 0.03 nm, and the SMSR was more than 25 dB. It can be continuously tuned to about 48 nm (1526.02~1573.91 nm), and the power change in the tuning range was only 1.46 dB.

In 2020, Lindberg et al. [56] reported a new tunable laser. The laser used a SOA as the gain medium and modulator and a chirped FBG as the end mirror at both ends of the cavity. It was continuously tuned at 35 nm (1535~1570 nm) with a linewidth less than 0.04 nm. Wang et al. [57] reported a tunable laser based on chirped FBG. The laser was tuned continuously for 30 nm (1535~1565 nm), with a SMSR greater than 50 dB and linewidth less than 8.5 kHz.

In the same year, Gao et al. [58] reported a tunable dual-wavelength fiber ring cavity laser based on FBG and externally injected distributed feedback (DFB) lasers. The wavelength spacing was adjusted by adjusting the operating temperature of the DFB laser. The laser used SOA as the gain material. When the FBG was operated at 40 °C, the DFB laser was operated at 10 °C, and the input current of the SOA was 120 mA. It was observed that the stable dual-wavelength simultaneous oscillation was achieved at 1550.32 nm and 1552.40 nm, and the wavelength spacing was 2.08 nm. The threshold current of 1550.32 nm and 1552.40 nm laser was about 98 mA and 25 mA. When the operating temperature difference between the FBG and the injected DFB laser varies between 30 °C and 0 °C, the wavelength spacing was adjusted from 2.08 nm to 5.34 nm.

In 2021, Congar et al. [59] reported an InGaN SL based on FBG with an emission wavelength of 400.5 nm and demonstrated its high coherence. The SMSR was 44 dB, the linewidth was 16 kHz and the output power was 1 mW. In the same year, the team demonstrated a compact and robust design based on a low-cost blue SL, beam-shaping optical system and FBG as a wavelength selective reflector. The design allowed for mW-level power output around 400.8 nm, when the SMSR approached 50 dB [60]. Monga et al. [61] reported a laser composed of FBG to improve the stability of the output power and narrow linewidth. The central wavelength of the laser was 1550 nm, the SMSR was 82 dB and the linewidth was 16 kHz.

In 2022, Su et al. [62] reported a frequency-stable narrow linewidth FBG-ECSL for carbon dioxide detection Lidar. The FBG-ECSL module was integrated in a compact butterfly package with an emission wavelength of 1572.02 nm, continuous tuning at 22 GHz (about 0.173 nm) and SMSR greater than 50 dB. The output power was 30 mW at an injection current of 340 mA. A polarization-preserving all-fiber ring resonator was used as the transmission cavity, and the linewidth of the ECSL was 15 kHz by combining the narrow linewidth technology with the frequency stable transfer technology.

Similar to VBG-ECSLs, in addition to universities and research institutes, there are also many photoelectric companies conducting commercial research on FBG-ECSLs, including Denselight in Singapore, WL Photonics in Canada, iXblue in France and others.

Denselight Singapore, founded in 1998, is an international optoelectronic chip optoelectronic device manufacturer. Its products cover 800~1700 nm and power up to 50 mW. Denselight also produces a number of ultra-narrow linewidth lasers for use in BOTDR or OTDR systems.

Ixblue (French) is a professional manufacturer of optical communication equipment, which owns a number of high-tech R&D and production companies, such as Photline and iXfiber. Photline was founded in 2000 and originated from the Optical Laboratory of the University of Besancon in France. The laboratory is a specialized integrated optics and optoelectronics research center in France. The product line includes strength modulator, phase modulator, 800~2000 nm modulator products, a variety of supporting RF drivers, bias voltage controllers and so on. IXfiber is the world’s leading active and passive specialty optical fiber manufacturer, providing various industries with components and modules based on raster technology for applications in the fields of sensors, telecommunications, military and aerospace. Its main products include: single-mode fiber, multi-mode fiber, special fiber, Bragg grating and so on. Take the 15XX series FBG lasers produced by iXfiber as an example, which can be divided into low-noise and high-power FBG lasers. The output wavelength of the low-noise FBG laser is 1530~1565 nm, the linewidth is 1 kHz and the output power is only 0.05 mW. The output wavelength of the high-power FBG laser is 1535~1565 nm, the output power is 10 mW, but the linewidth is 30 kHz.

Germany Innolume GmbH mainly produces quantum well semiconductor lasers and laser modules. First engaged in GAAS-based LD research, the wavelength can cover 780~1340 nm and can now provide almost any kind of laser diode chip between 780 and 1340 nm. The company’s 1064 nm FBG laser has an output power of 670 mW, SMSR > 15 dB and linewidth < 0.5 nm.

WL Photonics (Canada) designs, develops, manufactures and markets innovative photonics products for optical fiber wavelength tuning and tuning solutions for the optical communications, spectral instrumentation and fiber sensing industries. The company’s main products include: fiber phase compensators, tunable fiber lasers, FBG lasers, wavelength scanning lasers and portable spectral analyzers. In the case of the WLTL series, tunable fiber Bragg grating lasers are manufactured on the basis of a patented design. Each component can be configured according to the specific requirements to achieve a specific tuning range and output power, involving X, O, S, C and L bands in the field of optical communication. The central wavelengths of the series include 1060 nm, 1310 nm and 1550 nm; the tuning range is greater than 85 nm; the maximum tuning range is 100 nm; the output power is greater than 5 mW and the linewidth is less than 0.05 nm. It has a wider tuning range, narrower linewidth, wider involving frequency band range and lower noise, suitable for dense wavelength division multiplexing (the DWDM system uses multiple wavelengths for communication, greatly improving the transmission capacity of the system).

Nolatech was founded in 1992, and the main products include: a laser diode, FBG laser, DFB laser, tunable laser and so on. The 790 nm FBG laser designed by the company has a linewidth of less than 100 kHz and can be continuously tuned to 0.3 nm, which is suitable for optical measurement, optical communication and other fields.

The research progress of FBG-ECSLs in recent years is shown in Table 2. FBG is similar to VBG in that the linewidth of narrow lasers can be compressed more effectively, even to the sub-kHz order, by introducing FBG into the outer cavity. In addition to reducing the linewidth, FBG can also stabilize the center wavelength of the FBG-ECSLs, which can be used for continuous or quasicontinuous output. As can be seen from Table 2, the linewidths of the FBG-ECSLs studied in most laboratories are within the range of kHz~GHz, the SMSR is greater than 40 dB and the tuning range is in the order of GHz or THz (from tens of PI meters to tens of nanometers; in contrast, the tuning range is better than that of VBG-ECSLs). The narrowest linewidth can be as low as 125 Hz while having a continuous tunable range of 0.8 nm. The maximum SMSR is 82 dB with a narrow linewidth of 16 kHz. The maximum continuous tuning range is 48 nm. The linewidth of commercial FBG-ECSLs developed by the company is generally in the range of kHz~GHz, and the tuning range is in the order of GHz~THz. The narrowest linewidth is 1 kHz and can be continuously tuned to 35 nm, but the output power is only 0.05 mW. The widest tuning range is 85 nm, but the linewidth is on the order of GHz and the power is only 5 mW. Compared with laboratory results, most commercial FBG-ECSLs also have a larger tuning range and output power, but a smaller SMSR and a relatively wider linewidth. FBG-ECSLs are generally composed of a SL chip, FBG and piezoelectric transducer, which has the advantages of a simple and compact structure, narrow linewidth, good stability and certain tuning ability, and the production cost is low, the manufacturing is more mature and the uniformity of grating structure can be precisely controlled. The wavelength selectivity of FBG has also been used to develop other devices, such as mode-locked ECSL, filters and optical switches. At the same time, FBG also has a high SMSR. FBG is usually attached to a piezoelectric transducer or other elastic element (such as a bendable cantilever) to adjust the output wavelength (with a small tunable range) by applying strain to the FBG. Due to the small refractive index and large size of FBG material and its own material absorption loss, it is not conducive to improving the output power of ECSL. In general, FBG-ECSLs are suitable for a narrow linewidth, but the tuning range and power requirements are not high. Therefore, FBG-ECSLs can be widely used in optical fiber sensing, spectral gas sensing, optical coherence tomography and other fields and has potential applications in underwater optical communication, microwave photonics, spaceborne carbon dioxide detection Lidar, ground–starlight doppler ranging and so on.

### 3.3. WBG-ECSLs

**Figure 7:**
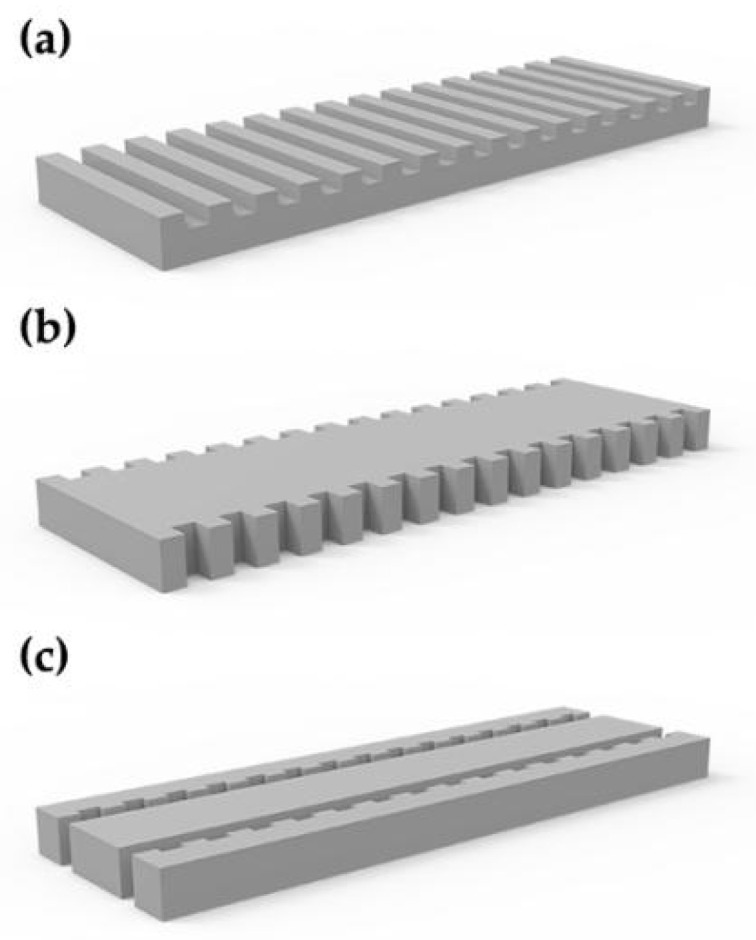
Structures of WBG: (**a**) surface WBG, (**b**) side wall WBG and (**c**) cladding WBG.

WBG has many advantages over FBG. It can make use of the functions of dielectric materials, such as electro-optic effect, acousto-optic effect and birefringence of dielectric materials. The polarization of WBG-ECSL is generally more stable than that of FBG-ECSL. In addition, the coupling is more stable than that between SL and fiber. Similar to FBG, WBG is a periodic structure added into a straight waveguide. Figure 7 shows the WBG structure.

**Figure 8:**
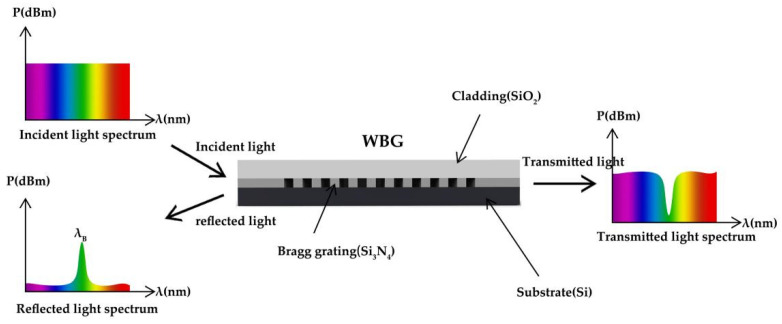
WBG reflection principle.

WBG is a structure in which the refractive index of the optical waveguide changes periodically, and the effective refractive index of the waveguide changes periodically through the thickness of the waveguide layer. At the periodic change, light reflection will be triggered, and light interference will be generated between the reflected light waves. By adding periodic modulation to the incident light field, light reflection of a specific wavelength is realized. Figure 8 is the reflection principle of WBG.

**Figure 9:**
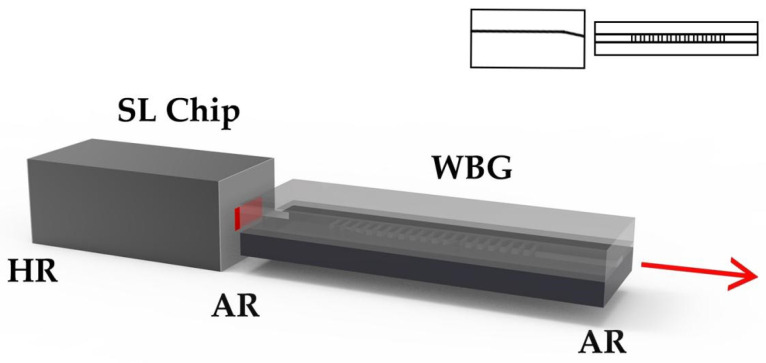
Structure of WBG-ECSL.

WBG-ECSL is a device that couples WBG with a SL chip, and its structure is shown in Figure 9, where the gain chip is a SL chip or SOA chip and where one cavity surface is highly reflectively (HR) coated, and the other surface is antireflective (AR) coated. The output of the waveguide is collimated through a lens, or coupled to a tail fiber. The basic materials of SL are III–V compounds, such as AlGaAs, InGaAs, InGaAsP, etc., while waveguides can choose various materials different from the chip, including semiconductors and dielectric materials.

In 2010, Numata et al. [68] reported a WBG-ECSL. The laser had a wavelength of 1542.383 nm and continuously tuned range was 47 GHz (about 0.37 nm). The maximum output power was 15 mW at an injection current of 180mA. In the same year, Yan et al. [69] combined waveguides with internal distributed Bragg gratings to reduce the linewidth (GHz to sub-GHz narrow linewidth was achieved).

In 2011, Oh et al. [70] reported a tunable WBG-ECSL, which consisted of a superlight-emitting diode and a WBG. The maximum output power was 12.2 mW, and the slope efficiency was about 0.15 W/A. The continuous tuning range was 20 nm (1527~1547 nm), linewidth was less than 0.3 nm and SMSR was greater than 35 dB. In the same year, Kyung-jo et al. [71] reported a WBG-ECSL operating at near-infrared wavelengths. The output wavelength of the laser was 850 nm, and the SMSR was 40 dB.

In 2012, Son et al. [72,73] implemented and optimized a near-infrared wavelength WBG-ECSL. The laser was tuned continuously at 20.8 nm (827.9~848.7 nm), the linewidth was 0.2 nm and the SMSR was greater than 40 dB. In the same year, Kotelnikov et al. [74] reported a 1550 nm WBG-ECSL. The output power of the laser reached 200 mW at an injection current of 1 A. The linewidth was 0.15 nm, and the SMSR was greater than 15 dB.

In 2013, Kim et al. [75] used WBG to study near-infrared wavelength tunable lasers with a central wavelength of 830 nm. The laser was tuned continuously to 31.7 nm (830.7~862.4 nm), and the SMSR was greater than 35 dB. In the same year, Kim et al. [76] designed an external cavity laser with a wide tuning range by combining a flexible polymer waveguide with a Bragg reflection grating. The laser had a tuning range of 81.8 nm (1500.1~1581.9 nm), the linewidth was less than 0.1 nm and the SMSR was 43 dB.

In 2014, Numata et al. [77] reported a 1064 nm WBG-ECSL. The WBG-ECSL was composed of a gain chip, a WBG and a prism and was tuned in the continuous range of 77 GHz (1064.36~1064.65 nm). The output power was 15 mW at a 95 mA injection current. In the same year, Lynch et al. [44] reported a 1649 nm WBG-ECSL using SL chips, WBG and other devices. The Bragg grating and waveguide were simultaneously written into the photosensitive core layer of the device by UV writing technology. The device had a linewidth of less than 1.1 GHz, SMSR was greater than 55 dB, the slope efficiency was 0.008 W/A, the threshold was 130 mA and a continuous tunable range was 690 pm. The output power was 0.5 mW at an injection current of 200 mA.

In 2015, Yun et al. [78] reported a WBG-ECSL. WBG was used as the wavelength selection element to adjust the wavelength by controlling the electric power on the microheater. The SMSR was 55 dB, the linewidth was 2 MHz, and continuously tunable range was 8.4 nm. In the same year, Mueller et al. [79] reported a WBG-ECSL. The tunable continuously range was 20 nm (1541~1561 nm), SMSR was greater than 35 dB. Lench et al. [80] report a WBG-ECSL. The central wavelength of the laser was 1650 nm and the linewidth was 200 kHz.

In 2016, Girschikofsky et al. [81] reported Bragg grating in waveguide of hybrid polymers using interferometric phase masks combined with deep ultraviolet laser radiation. The WBG with reflectivity up to 98% and linewidth was less than 120pm. In the same year, Park et al. [82] reported a C-band tunable WBG-ECSL. The center wavelength was 1542 nm, the SMSR was 23.5 dB, and tunable continuous range was 10 nm.

In 2017, Liu et al. [83] reported a new planar WBG structure. The structure consisted of two adjacent transverse gratings with slightly different grating periods. In addition, manufacturing costs are further reduced. The results showed that this structure was beneficial to improve the performances of grating based optical integrated devices.

In 2018, Iadanza et al. [84,85] reported a heat-stabilized silicon nitride ECSL. The laser was a combination of SOA and WBG with a power output of more than 3 mW at injection current of 100 mA. The linewidth was less than 3 MHz, and the SMSR was greater than 40 dB.

In 2019, Primiani et al. [86] reported a WBG-ECSL with a central wavelength of 1543 nm based on the Bragg gratings and SOA produced by the silicon nitride process. By changing the bias current of the SOA, 0.16 nm was tuned continuously. The linewidth was less than 17 kHz, and the SMSR was more than 60 dB. In the same year, Xiang et al. [87] reported an ultra-narrow linewidth laser consisting of an SL chip and a silicon nitride Bragg grating. The central wavelength of the laser was 1544 nm, and the linewidth was 320 Hz.

In 2020, Luo et al. [88] reported a C-band birefringent WBG using displacement tachyon lithography. By adjusting the waveguide width and grating etching depth, the birefringence of WBG can be controlled. Compared with WBG of other platforms, the mode splitting between TE mode and TM mode of the device reached 1.7635 nm. It had excellent function of wavelength selection and polarization mode selection.

In 2021, Janjua et al. [89] reported for the first time a narrow linewidth edge emitting semiconductor Bragg reflection waveguide laser. The SMSR of the laser was 49 dB and the linewidth was 207 kHz. The output power was 2.5 mW at injection current of 100 mA.

In the same year, Ibrahimi et al. [90] reported a WBG-ECSL. The output power was 7.5 mW at injection current of 300 mA. Wang et al. [91] reported a monolithic integrated narrow linewidth WBG-ECSL. It was composed of an active part and a passive narrow-band reflector based on WBG, which can obtain a narrow linewidth of about 16 kHz. XI-Luo et al. [92] reported a narrow linewidth hybrid laser based on a SL gain chip and a high birefringence WBG composition. The laser operated in the C-band with a central wavelength of about 1552 nm. The linewidth was 4.15 kHz and the SMSR was up to 52 dB.

In 2022, Zheng et al. [93] reported the influence of various factors on the performances of WBG. The results showed that the minimum linewidth of the WBG was 0.1 nm, the maximum SMSR was 13.2 dB, and the maximum reflectance was 99%. In the same year, Luo et al. [94] reported a narrow linewidth hybrid laser based on a SL gain chip and birefringent silicon WBG. The optimal linewidth was 4.36 kHz and the output power was 6.53 mW.

In addition to universities and research institutes, there are many companies developing commercial products of WBG-ECSLs, including Toptica in Germany, QD Laser in Japan, Finisar in the United States, etc.

Toptica (Germany) develops and manufactures high-end laser systems for scientific and industrial applications. The portfolio includes diode lasers, ultrafast fiber lasers, terahertz systems and frequency combs. Not only that, the company’s diode lasers have excellent coherence, wide tuning ranges and ideal beam profiles. The company has designed a 760 nm laser with a power output of up to 80 mW and can be continuously tuned to 33 nm. The 980 nm laser has an output power of 50 mW and can be continuously tuned to 100 nm.

Finisar is the world’s largest and most advanced supplier of optical components, modules and subsystems for telecommunications equipment and service providers, optical displays, security systems, medical devices, environmental protection equipment, aviation and defense systems. The company’s laser tuning range covers the entire C-band, suitable for DWMD systems, optical switches and other fields.

QDLaser is a leading manufacturer of semiconductor lasers for the telecommunications industry and displays. The company can use nanoscale semiconductor particles of quantum dots to achieve properties not normally available in standard lasers. Not only that, QDLaser provides laser diodes in the 1 μm to 1.3 μm range for CW or pulse mode. The company produces 1064 nm semiconductor laser, the output power of more than 100 mW.

GLSUN (China), founded in 2001, is a high-tech enterprise specializing in the development, production and sales of high-end semiconductor laser chips and components, active and passive optical devices. The 1310 nm laser produced by the company can be tuned to 20 nm and the output power is 40 mW, which is suitable for data communication, optical transmission and other fields.

Germany Photonics produces tunable lasers with output power exceeding 16 dBm, linewidth < 25 kHz, and continuous tuning of 41 nm in the C and L bands.

RIO is the leading global supplier of single frequency narrow linewidth lasers, modules, and subsystems to the clean energy, security, oil and gas, and test and measurement markets. It’s lasers have ultra-low noise, very narrow linewidth, unparalleled wavelength stability, small size, low power dissipation, Telecom grade lifetime reliability, at affordable prices. The company’s 1550 nm laser has a linewidth of less than 1 kHz, an output power of 20 mW and can be continuously tuned to 35 nm.

The research progress of WBG-ECSLs in recent years is shown in Table 3. As can be seen from Table 3, the linewidth of WBG-ECSLs studied in most laboratories is within the range of kHz~GHz, and the SMSR is greater than 35 dB (but some SMSRs are very small, only more than ten dB). The tuning range is in the order of GHz, THz (from hundreds of pimmeters to tens of nanometers can not, in contrast, the tuning range is better than FBG-ECSLs). The narrowest linewidth can be as low as 320 Hz, and the SMSR can be greater than 55 dB. The maximum SMSR is greater than 60 dB, and the linewidth is less than 17 kHz, which can be continuously tuned to 20.2 GHz. The maximum tuning range is 81.8 nm. The linewidth of commercial WBG-ECSLs developed by the company is generally in the order of kHz~MHz, from tens of kHz to several MHz, SMSR is greater than 35 dB, and the tuning range is in the order of THz (tens of nm to hundreds of nm). The narrowest linewidth is 1 kHz and can be continuously tuned to 35 nm, but the output power is only 0.05 mW. The maximum tuning range is 100 nm and the linewidth is <6.2 GHz. In contrast, the laboratory product linewidth characteristics are better, but the tuning capability and output power are relatively low, while the commercial WBG-ECSLs have good tuning range, linewidth characteristics and output power. It can be seen that compared with VBG-ECSLs and FBG-ECSLs, WBG-ECSLs has excellent comprehensive performance, including narrow linewidth characteristics, larger tuning range and SMSR. Moreover, due to the particularity of waveguide materials (Si, SiO_2_, Si_3_N_4_) (good thermal stability), The center wavelength of WBG-ECSLS has a smaller shift with temperature, and its performance is more stable. Moreover, waveguide has the advantages of simple structure, small size, low noise and low production cost, which can be combined with CMOS technology for large-scale production. WBG-ECSLs is generally composed of SL chip, WBG, piezoelectric actuator and microheater. Through the selection of gain medium, material and integrated device, as well as the new external cavity structure design, the epitaxial design of SOA is improved, the structure of Bragg waveguide is optimized, the coupling efficiency is improved, the waveguide loss is reduced, and the reflectivity is adjusted. Thus, WBG-ECSLS with narrower linewidth (sub kHz magnitude), wider tuning range, higher SMSR, lower noise, low cost, small volume and high performance can be realized. In general, with the continuous maturation of silicon chip design and process platform, the types of external cavity feedback components based on SiO_2_, Si_3_N_4_, and other materials have become more diverse. By introducing WBG structure, the linewidth of WBG-ECSLs can be compressed and the coherence of output can be improved. In addition, the wavelength tuning range of WBG-ECSLs can be significantly increased. At the same time, WBG-ECSLs has higher integration and lower loss, which can take into account narrow linewidth and tunability. WBG-ECSLs can be widely used in gas detection, coherent optical communication, wavelength division multiplexing system and other fields, and has potential application prospects in high-precision spectral detection, biomedical imaging system, high-speed long-distance quantum communication system and so on.

## 4. Conclusions

BG-ECSLs can be widely used in optical communication, gas detection, coherent light detection, synthetic aperture Lidar, spectral gas sensing and other fields and has potential application prospects in underwater optical communication, spaceborne carbon dioxide detection Lidar, biomedical imaging system, high-speed long-distance quantum communication system, etc. [103,104,105]. In this paper, the principle of BG-ECSLs is described, and the research achievements and latest progress in this field in the past ten years are reviewed. As shown in Table 4, the linewidth of VBG-ECSLs is in the range of kHz-THz (generally in the order of kHz-GHz), the SMSR is in the range of 16~57 dB and the tuning range is in the order of GHz (most of them are tens of picometers). The narrowest linewidth can be as low as 2 kHz while having SMSR up to 57 dB, but the continuous tunable range is small, only 0.063 nm. The maximum tuning range is 1.9 nm. The linewidth of FBG-ECSLs is in the range of kHz~GHz and that of SMSR is in the range of 25~82 dB. The tuning range is in the order of GHz and THz (from tens of picometers to tens of nanometers, and the tuning range is better than that of VBG-ECSLs). The narrowest linewidth can be as low as 125 Hz while having a continuous tunable range of 0.8nm. The maximum SMSR is 82 dB with a narrow linewidth of 16 kHz. The maximum continuous tuning range is 48 nm. The linewidth of WBG-ECSLs is in the range of KHz~GHz, and that of SMSR is in the range of 15~82 dB. The tuning range is in the order of GHz and THz (from hundreds of picometers to tens of nanometers, the tuning range is better than FBG-ECSLs). The narrowest linewidth can be as low as 320 Hz, and the SMSR can be greater than 55 dB. The maximum SMSR is greater than 60 dB, and the linewidth is less than 17 kHz, which can be continuously tuned to 20.2 GHz. The maximum tuning range is 81.8 nm. From the characteristics of commercial BG-ECSLs products, it is not difficult to find that VBG-ECSLs can obtain relatively large output power, while FBG-ECSLs and WBG-ECSLs can obtain a relatively wide tuning range, and the tuning range of WBG-ECSLs is larger, because commercial products are highly targeted (most of them are aimed at a certain module in a certain application or even a certain band). In order to meet the parameter requirements of their application field, most companies do not pursue performances with a low parameter impact on the field. For the application of DWDM, the company pursues parameters such as tuning range, linewidth, output power, SMSR, etc. when conducting research on commercial products, but some parameters cannot obtain the optimal values at the same time. For example, according to Formula (2), if the linewidth is narrower, the output power will be lower. Therefore, in the design of products, it is necessary to meet the requirements of the primary parameters (tuning range), combined with the existing technology, comprehensive consideration of production costs, product stability and other factors to adopt the optimal choice.

The advantages of VBG-ECSLs are the simple structure of the outer cavity and the lack of moving parts, thus providing maximum mechanical stability and reliability. and the center wavelength of the outer cavity structure can be adjusted by tilting the angle of the VBG or changing the cavity length. It plays an important role in biomedicine, optical data storage, synthetic aperture Lidar, quantum optical sensors and other fields. FBG-ECSLs can compress the linewidth of narrow lasers more efficiently, even to the sub-kHz magnitude. It has the advantages of simple and compact structures, narrow linewidths, good stability and a certain tuning ability, and the production cost is low, so the manufacturing process is more mature. However, due to the small refractive index, the large size and large material absorption loss of FBG material itself, it is not conducive to improving the output power of ECSL. Therefore, FBG-ECSLS is suitable for a narrow linewidth, but the tuning range and power requirements are not high. It can be widely used in optical fiber sensing, spectral gas sensing, optical coherence tomography and other fields and has potential application prospects in underwater optical communication, microwave photonics, spaceborne carbon dioxide detection Lidar, ground–starlight Doppler ranging and so on. WBG-ECSLs can not only narrow the linewidth but also maintain good SMSR and tuning capability. Compared with VBG-ECSLs and FBG-ECSLs, WBG-ECSLs have excellent comprehensive performances, which not only have narrow linewidth characteristics but can also maintain a relatively large tuning range and SMSR. Moreover, due to the particularity (good thermal stability) of waveguide materials (Si, SiO_2_ and Si_3_N_4_), WBG-ECSLs has a smaller central wavelength offset with the temperature. The work performance is more stable. Moreover, waveguide has the advantages of a simple structure, small size, low noise and low manufacturing cost, which can be combined with CMOS technology for large-scale production. Through the selection of gain media, materials, integrated devices, new external cavity structure design and optimization of Bragg waveguide structure, so as to improve the coupling efficiency and reduce the waveguide loss, so as to achieve a narrower linewidth, wider tuning range, higher SMSR, lower noise, low-cost and small-volume performance WBG-ECSLs. In general, with the continuous maturation of silicon chip designs and process platforms, the types of external cavity feedback components based on SiO_2_, Si_3_N_4_ and other materials have become more diverse. By introducing the WBG structure, the linewidth of WBG-ECSLs can be compressed, and the coherence of the output can be improved. In addition, the wavelength tuning range of WBG-ECSLs can also be significantly increased. WBG-ECSLs can be widely used in gas detection, coherent optical communication, wavelength division multiplexing system and other fields and have potential applications in multicomponent detection, multi-atom cooling, high-precision spectral detection, biomedical imaging system, high-speed long-distance quantum communication system and so on.

At present, due to the late start and technical blockade, the research on BG-ECSLs still has a certain gap with the international top level. Therefore, the key technologies (epitaxial growth, etching, etc. [106]) should be continuously broken through in the future research, and the device structure should be optimized [107]. The internal cavity (gain chip) optimizes the chip structure. The external cavity (Bragg grating and other optical feedback components [93,108,109]) adjust the structure of the existing optical components and design the optical feedback components with a better performance. In general, WBG-ECSLs are a potential approach for laser output with integration, small size, narrow linewidth, wide tuning range, stable spectral output and high side-mode rejection ratio. Using WBG as an optical feedback element is still the mainstream direction of BG-ECSLs.

## Figures and Tables

**Table 1:** The performances parameters of VBG-ECSLs.

Type	λ_B_	Linewidth	SMSR	Tuning Range		Current	Power	Year
VBG	1179.9 nm 1182.8 nm	84 GHz *	>34 dB	-	-	1 A	0.186 W	2010 [12]
VBG	810 nm	19 MHz	37 dB	0.072 nm	32.9 GHz *	-	10 mW	2011 [13]
VBG	633 nm	<10 MHz	>25 dB	0.034 nm	25 GHz *	46 mA	5.3 mW	2012 [15]
VBG	780.24 nm	2 kHz	>57 dB	0.063 nm *	31 GHz	250 mA	123 mW	2013 [17]
VBG	766.7 nm	3 kHz	>45 dB	0.054 nm*	27.5 GHz	240 mA	33.4 mW	2014 [18]
VBG	780 nm	36 kHz	>50 dB	0.011 nm *	5.2 GHz	535 mA	380 mW	2015 [19]
VBG	1064 nm	8.7 GHz *	-	0.11 nm	29 GHz *	3 A	1.3 W	2015 [20]
VBG	445 nm	1.5 THz *	50 dB	-	-	1.2 A	1.4 W	2016 [21]
VBG	767 nm	55 kHz	-	0.098 nm *	50 GHz	200 mA	30 mW	2016 [23]
VBG	1064.49 nm	30 kHz	>45 dB	-	-	100 mA	4 mW	2017 [24]
VBG	808.07 nm	124 GHz *	26 dB	1.9 nm *	0.88 THz	2.5 A	0.415 W	2018 [25]
VBG	708.24 nm	100 kHz	54 dB	-	-	-	50 mW	2019 [28]
VBG	780.25 nm	19 kHz	-	0.016 nm *	8 GHz	150 mA	30 mW	2020 [29]
VBG	808 nm	2.8 GHz *	-	-	-	6 A	4.3 W	2020 [30]
VBG	976 nm	157 GHz *	>30 dB	-	-	50 A	33.9 W	2021 [32]
VBG	405.1 nm	146 GHz *	-	0.06 nm	109.7 GHz *	300 mA	292 mW	2022 [33]
VBG	762 nm	2.1 GHz *	16 dB	0.012 nm	6.2 GHz *	3 A	1.31 W	2022 [35]
VBG	780 nm	70 kHz	-	0.018 nm *	9 GHz	250 mA	30 mW	2022 [36]
VBG	811.53 nm	68.3 GHz *	40 dB	0.2 nm	91.1 GHz *	10 A	106.4 W	2022 [37]
VBG	808 nm	229.8 GHz *	-	1.2 nm	551.4 GHz *	<70 A	40 W	2022 [38]
VBG	830 nm	43.5 GHz *	40 dB	1 nm	435.5 GHz *	1.5 A	0.8 W	2022 [39]
VBG	1064 nm	<100 kHz	>35 dB	1 nm	265 GHz *	350 mA	50 mW	2022 [40]
VBG	785 nm	29.2 GHz *	>40 dB	>0.125 nm	>60.9 GHz *	1.5 A	0.6 W	2022 [41]
VBG	976 nm	314.9 GHz *	>40 dB	2 nm	629.9 GHz *	9.5 A	100 W	2022 [42]

Note: “*” denotes that the data is calculated, and “-” denotes that the data is not available.

**Table 2:** The performances parameters of FBG-ECSLs.

Type	λ_B_	Linewidth	SMSR	Tuning Range		Current	Power	Year
FBG	760 nm	<208 GHz *	>50 dB	1 nm	519.4 GHz *	-	-	2010 [43]
FBG	980 nm	<312 GHz *	>45 dB	0.1 nm	31.2 GHz *	400 mA	200 mW	2011 [44]
FBG	1550 nm	1 kHz	-	1.1 nm	146.7 GHz *	4 A	0.37 W	2011 [45]
FBG	1648.2 nm	<5 MHz	-	-	-	500 mA	3.5 mW	2012 [46]
FBG	974 nm	<31.6 GHz *	>45 dB	-	-	100 mA	7.1 mW	2014 [47]
FBG	1035 nm	67.2 GHz *	50 dB	0.1 nm	28 GHz *	-	400 mW	2014 [48]
FBG	1647 nm	55.3 GHz *	-	1 nm	110.6 GHz *	-	-	2014 [49]
FBG	1532.83 nm	<14 kHz	>60 dB	-	-	350 mA	9 mW	2016 [50]
FBG	1549.7 nm	125 Hz	-	0.8 nm	99.9 GHz *	131 mA	3 mW	2016 [51]
FBG	1550 nm	<3 kHz	-	0.173 nm *	21.6 GHz	270 mA	20 mW	2016 [52]
FBG	1550 nm	35 kHz	>50 dB	0.5 nm	62.4 GHz *	187 mA	-	2017 [53]
FBG	976 nm	<50.4 GHz *	>40 dB	1.0925 nm	344.1 GHz *	100 mA	26.5 mW	2018 [54]
FBG	1550 nm	<3.7 GHz *	>25 dB	48 nm	6 THz *	195 mA	400 mW	2019 [55]
FBG	1550 nm	5 GHz *	>35 dB	35 nm	4.7 THz *	480 mA	-	2020 [56]
FBG	1550 nm	8.5 kHz	>50 dB	30 nm	3.7 THz *	39 mA	13 mW	2020 [57]
FBG	1550.32 nm 1552.40 nm	-	-	3.26 nm	407.1 GHz *	150 mA	0.48 mW	2020 [58]
FBG	400.5 nm	16 kHz	44 dB	0.5 nm	935.2 GHz *	95 mA	1.3 mW	2021 [59]
FBG	400.8 nm	56.3 GHz *	46 dB	0.5 nm	933.8 GHz *	110 mA	5.3 mW	2021 [60]
FBG	1550 nm	16 kHz	82 dB	-	-	-	12 mW	2021 [61]
FBG	1572.02 nm	15 kHz	>50 dB	0.181 nm *	22 GHz	340 mA	30 mW	2022 [62]
FBG	1550 nm	<200 kHz	>45 dB	4 nm	499.5 GHz *	100 mA	10 mW	2022 [63]
FBG	1550 nm	1 kHz 30 kHz	-	35 nm 30 nm	4.4 THz * 3.7 THz *	-	0.05 mW 10 mW	2022 [64]
FBG	1064 nm	<132.5 GHz *	>15 dB	5 nm	1.3 THz *	1 A	0.67 W	2022 [65]
FBG	1060 nm	<13.3 GHz *	-	85 nm	22.7 THz *	-	5 mW	2022 [66]
FBG	790 nm	<100 kHz	>40 dB	0.3 nm	144.2 GHz *	200 mA	20 mW	2022 [67]

Note: “*” denotes that the data is calculated, and “-” denotes that the data is not available.

**Table 3:** The performances parameters of WBG-ECSLs.

Type	λ_B_	Linewidth	SMSR	Tuning Range		Current	Power	Year
WBG	1542.383 nm	-	-	0.37 nm *	47 GHz	180 mA	15 mW	2010 [68]
WBG	1537 nm	<38.1 GHz *	>35 dB	20 nm	2.5 THz *	60 mA	9 mW	2011 [70]
WBG	838.8 nm	85.3 GHz *	>40 dB	20.8 nm	8.9 THz *	55 mA	1.5 mW	2012 [72]
WBG	1550 nm	18.7 GHz *	>15 dB	-	-	1 A	0.2 W	2012 [74]
WBG	830 nm	87.1 GHz *	35 dB	31.7 nm	13.8 THz *	-	-	2013 [75]
WBG	1535 nm	12.7 GHz *	43 dB	81.8 nm	10.4 THz *	-	-	2013 [76]
WBG	1064 nm	-	-	0.29 nm	77 GHz *	95 mA	15 mW	2014 [77]
WBG	1649 nm	<1.1 GHz	>55 dB	0.69 nm	76.1 GHz *	200 mA	0.5 mW	2014 [49]
WBG	1546 nm	2 MHz	55 dB	8.1 nm	1 THz *	-	-	2015 [78]
WBG	1551 nm	-	>35 dB	20 nm	2.5 THz *	75 mA	20 mW	2015 [79]
WBG	1650 nm	200 kHz	-	-	-	400 mA	3.9 mW	2015 [80]
WBG	1542 nm	-	23.5 dB	10 nm	1.3 THz *	-	312 mW	2016 [82]
WBG	1556 nm	<3 MHz	>40 dB	0.744 nm	92.2 GHz *	100 mA	3 mW	2018 [84]
WBG	1543 nm	<17 kHz	>60 dB	0.16 nm	20.2 GHz *	200 mA	15 mW	2019 [86]
WBG	1544 nm	320 Hz	>55 dB	-	-	92 mA	24 mW	2019 [87]
WBG	795 nm	207 kHz	49 dB	9 nm	4.3 THz *	100 mA	2.5 mW	2021 [89]
WBG	1550 nm	31.2 GHz *	10 dB	-	-	300 mA	7.5 mW	2021 [90]
WBG	1552 nm	4.15 kHz	52 dB	1.62 nm	201.8 GHz *	400 mA	8.07 mW	2021 [92]
WBG	1550 nm	4.36 kHz	>49 dB	0.6 nm	80 GHz *	208 mA	6.53 mW	2022 [94]
WBG	980 nm	-	-	100 nm	31.2 THz *	100 mA	50 mW	2022 [95]
WBG	1528.8 nm	5 MHz	>40 dB	40.8 nm	5.2 THz *	240 mA	20 mW *	2022 [96]
WBG	1064 nm	-	40 dB	10 nm	2.6 THz *	200 mA	120 mW	2022 [97]
WBG	1060 nm	-	-	40 nm	10.7 THz *	250 mA	80 mW	2022 [98]
WBG	760 nm	-	-	33 nm	17.1 THz *	180 mA	80 mW	2022 [99]
WBG	1310 nm	-	>35 dB	20 nm	3.5 THz *	150 mA	40 mW	2022 [100]
WBG	1550 nm	<25 kHz	>40 dB	41 nm	5.1 THz *	500 mA	60 mW *	2022 [101]
WBG	1550 nm	<1 kHz	40 dB	35 nm	4.4 THz *	-	20 mW	2022 [102]

Note: “*” denotes that the data is calculated, and “-” denotes that the data is not available.

**Table 4:** Comparison of different kinds of BG-ECSLs.

Type	VBG	FBG	WBG
Main Materials	Photo-Thermo-Refractive, Polymer	Glass, Crystal, Plastomer	Si, SiO_2_, Si_3_N_4_, LiNbO_3_, Polymer
Linewidth Range	2 kHz~1.5 THz	125 Hz~312 GHz	320 Hz~85.3 GHz
Min linewidth	2 kHz	125 Hz	320 Hz
SMSR Range	16 dB~57 dB	15 dB~82 dB	15 dB~60 dB
Max SMSR	57 dB	82 dB	60 dB
Tuning Range	0.011 nm~2 nm	0.1 nm~85 nm	0.16 nm~100 nm
Max Tuning Range	2 nm	85 nm	100 nm
Output Power	10 mW~106.4 W	0.05 mW~670 mW	0.5 mW~312 mW
Maximum Output Power	106.4 W	670 mW	312 mW

## Data Availability

Not applicable.
